# Probiotic isolates from unconventional sources: a review

**DOI:** 10.1186/s40781-016-0108-2

**Published:** 2016-07-19

**Authors:** Pairat Sornplang, Sudthidol Piyadeatsoontorn

**Affiliations:** Department of Veterinary Public Health, Faculty of Veterinary Medicine, Khon Kaen University, Khon Kaen, 40002 Thailand; Faculty of Agricultural and Technology, Rajamangala University of Technology Isan, Surin Campus, Surin, 32000 Thailand

**Keywords:** Probiotics, Unconventional sources, Lactic acid bacteria, Fermented food

## Abstract

The use of probiotics for human and animal health is continuously increasing. The probiotics used in humans commonly come from dairy foods, whereas the sources of probiotics used in animals are often the animals’ own digestive tracts. Increasingly, probiotics from sources other than milk products are being selected for use in people who are lactose intolerant. These sources are non-dairy fermented foods and beverages, non-dairy and non-fermented foods such as fresh fruits and vegetables, feces of breast-fed infants and human breast milk. The probiotics that are used in both humans and animals are selected in stages; after the initial isolation of the appropriate culture medium, the probiotics must meet important qualifications, including being non-pathogenic acid and bile-tolerant strains that possess the ability to act against pathogens in the gastrointestinal tract and the safety-enhancing property of not being able to transfer any antibiotic resistance genes to other bacteria. The final stages of selection involve the accurate identification of the probiotic species.

## Background

The term *probiotic* refers to live microorganisms that survive passage through the by improving its intestinal microbial balance [1, 2]. Recently, FAO/WHO has defined probiotics as living microorganisms that improve the health of humans and animals and must be safe and in sufficient quantity for bodily function [3]. For probiotic products to be identified as functioning, its concentration must be at least 10^6^ viable cells (colony forming unit, CFU/g) of the product. The discovery process for new probiotics emphasizes strain selection and the survival of the culture during biomass production and storage.

For at least the past 10 years, probiotic microorganisms have been used continuously for health benefits in both humans and animals. The main reason for their use is that probiotics offer an alternative to antibiotics; such an alternative is proposed to decrease the drug resistance that occurs due to an overuse or prolonged use of antibiotics to treat infections in both humans and animals. In addition, foods of animal origin have been found to contain drug residues in their meat, which is a result of animals being reared in conditions of antibiotic misuse, including an excessive use of antibiotics for disease treatment, incorrect drug withdrawal times and the addition of antibiotics to feed. When humans consume the contaminated foods, the drug accumulates in the body and leads to drug resistance when an antibiotic is used to treat an infection.

Gut microflora can also be balanced by directly adding live microorganisms into the diet. Microbes were used as probiotics including bacteria, yeast and mold. The genera and species that have been used are *Lactobacillus, Streptococcus*, *Leuconostoc, Pediococcus*, *Propionibacterium*, *Enterococcus*, *Bifidobacterium*, *Bacillus*, *Saccharomyces cerevisiae*, *Candida pintolopesii*, *Aspergillus niger* and *A. oryzae* [4]. Lactic acid bacteria (LAB) are considered a major group of probiotic bacteria and are commonly used in both humans and animals [5]. The most commonly used LAB in humans are *Lactobacillus* and *Bifidobacterium* [6]. Spore-forming lactic acid bacteria, mostly of the genus *Bacillus*, are acid and bile tolerant strains that have been used as probiotics in both humans and animals. However, only *Bacillus* strains that have not been reported to be pathogenic, such as *Bacillus lichenformis, B. cereus* var. *toyoi*, *B. clausii*, *B. coagulans, B. laterosporus, B. pumilus* and *B. racemilacticus* can be used as probiotics [7–9]. The studies and developments related to probiotics include research on selecting probiotic strains with specific properties and, technologies that can be used to improve the probiotic production process. Therefore, probiotic markets are likely to increase continuously.

### Unconventional sources of probiotics

The use of selected probiotics from alternative sources known as “unconventional sources” is likely to increase. One of the reasons why alternative sources for probiotic selection have increased in use is to avoid the consumption of dairy in lactose-intolerant individuals. Unconventional sources of microorganisms were screened for potential probiotics, which have been isolated from numerous different sources, including non-intestinal sources and non-dairy fermented food products, such as traditional fermented foods, traditional fermented drinks, vegetables, and fruit juices [10–13]. The differences in the raw materials and ingredients used to make non-fermented or fermented foods are the main factors that lead to the different available species or strains of probiotics in the food sources.

**Fig. 1:**
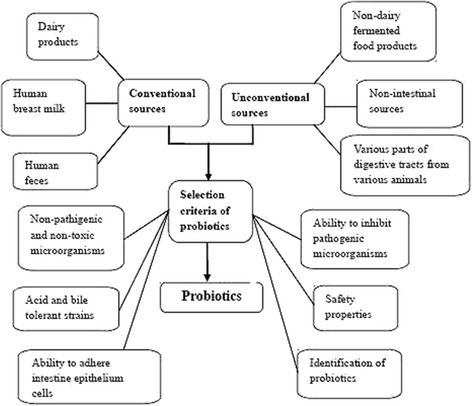
Diagram of probiotic sources and selection criteria to apply in both humans and animals

Probiotic microorganisms can be screened from non-intestinal sources, such as fruit juices [14], grains [15], honey-comb [16] and soil [17, 18]. Probiotic sources and selection criteria to apply in both humans and animals are summarized as the Fig. 1. LAB primarily *Lactobacillus plantarum* have been found in many types of fruit juices from both solid and citrus fruits whereas *Leuconostoc mesenteroides* is rarely found in these fruits but is the species that is most commonly found in tomatoes [14]. The alternative growth medium that is used to cultivate lactic acid bacteria can also be used to select probiotics from sources such as pineapple wastes [19] and tomato juice [20]. LAB can be found in food products stored at a low temperature (4 °C) such as vacuum-packaged beef and some beneficial isolates can be screened in a similar way as bacteria that produce bacteriocin-like substance [21].

Probiotics isolated from non-intestinal sources are likely to not be strains that produce a bacteriocin-like substance. For example, *Lactobacillus plantarum* isolated from fermented foods containing fish or pork were resistant to low pH, tolerated bile and exhibited strong antimicrobial activity against pathogenic bacteria when using the normal supernatant of the strain, whereas the neutralized supernatant showed weak antagonistic activity [22, 23]. Several fresh fruits and vegetables, including dragon, durian, ginger, papaya, star fruits and guava, can be screened to find beneficial LAB that produce antimicrobial substances other than bacteriocin, such as hydrogen peroxide and lactic and propionic acids, to combat against pathogenic bacteria and pathogenic fungi of chilies [22]. These LAB can be used for starter cultures in human fermented foods and can have probiotic effects in humans. However, some studies have reported that LAB from some fruits and vegetables showed bacteriocin-like activity [24]. Tajabadi et al. [16] have screened LAB from the honey of giant honey bees. Most of these LAB isolates were *Lactobacillus* spp., mainly *Lactobacillus kunkeei*. This bacterial species has been reported to have antagonistic effects against yeast growth and the spoilage-related effects of yeast in honey [25].

Siddiqee et al. [10] reported that animal intestines comprise the most potential sources of LAB but that other sources, including fruit juice, flesh, long grass and vegetables, can also be screened to find LAB. Milk whey as dairy waste from cheese production industries has been used to cultivate LAB to produce more lactic acid compared with the conventional Luria-Bertani medium. Thus, it may be the one of alternative sources from which beneficial LAB can be isolated [26].

Other unconventional sources from which high potential probiotics have been isolated are the environments around food products, such as the air surrounding environments for preparing sourdoughs and the air of the storage and working rooms of a bakery. These air samples have been found to contain *Lactobacillus plantarum*, a similar species to the species that was isolated from the dough [27].

### Probiotic isolates from non-dairy fermented foods

Fermented food products that are screened for potential probiotics are made from two main materials: plant and animal matter. Several types of fermented foods for human consumption differ in the materials and ingredients used to make them depending on the culture and traditions of the local people from each country. The difference in raw materials and ingredients used in fermented foods is a main factor that has led to the identification of different species and strains of probiotics. The various traditional fermented foods that have been found to contain LAB are made from a variety of raw materials, including fish [28, 29], beef [30], pork [31], salted crab [32], seafood [33], soybeans [34] and vegetables [12].

LAB species have been isolated from fermented foods from several countries. In Thailand, six genera of LAB species have been identified: *Aerococcus*, *Enterococcus*, *Lactobacillus*, *Pediococcus*, *Tetragenococcus* and *Weissella*. Most LAB genera isolated from fermented fishes and crustaceans is *Enterococcus*, while the LAB genera most commonly isolated from fermented meats and fermented plants is *Lactobacillus*. These strains are the most halophilic LAB and grew under a NaCl concentration of more than 22 %. However, the main species isolated from fermented plant materials are *L. fermentum* and *L. plantarum*. These species grew under NaCl concentrations of less than 6 % [35]. NaCl-like LAB has also been found in Thai traditional salted crab (*Poo-Khem*). Out of 306 isolates, four probiotic LAB species can be used as starter cultures in *Poo-Khem* foods for humans because they showed probiotic properties including acid and bile tolerance, antagonistic effects against food-borne pathogenic bacteria and hydrophobic activity. These four species were identified as one strain each of *Enterococcus thailandensis* and *L. plantarum* and two strains of *L. fermentum* [32]. In addition, Siripornadulsil et al. [31] reported that *Pediococcus pentosaceous* strains were the LAB most often isolated from various traditional Thai fermented foods containing fish and pork. They were tolerant to acidic conditions at pH 2, 0.3–0.5 % bile salt and 1–14 % NaCl. They also inhibited the growth of some pathogenic bacteria, including *Pseudomonas aeruginosa*, *Salmonella typhimurium*, *Vibrio cholera*, *E. coli*, *Bacillus cereus* and *Staphylococcus epidermidis*. Other beneficial effects of LAB, such as the ability to convert starch to lactic acid, have been observed in species isolated from Thai fermented rice noodles [36].

Probiotic *Pediococcus pentosaceous* strains are also the most commonly found strains in *Wakalim*, a traditional Ethiopian fermented beef sausage. These strains were tolerant to a pH of 3 and a 0.3 % bile salt concentration [37]. A fish sauce product is also a fermented food made from different raw materials such as fish and shellfish. It was found probiotic isolates such as *Lactobacillus plantarum*, *Saccharomyces cerevisiae* and *Staphylococcus arlettae*. They possessed inhibitory effect against *S. aureus* and *Listeria monocytogenes*. [38]. In India, *L. plantarum* isolated from fermented idli batter has been tested successfully as a co-aggregation with pathogens like *Listeria monocytogenes* and *Escherichia coli* [39]. In addition, LAB genera, including genera of *Enterococcus*, *Lactobacillus*, *Lactococcus*, *Vagococcus* and *Weissella*, have been isolated from a traditional fermented soybean food in India. These isolates, excluding *Vagococcus* sp. and *Weissella* sp., showed antibacterial activity against some pathogenic bacteria (*B. cereus*, *E. coli* and *Salmonella paratyphi*) [40]. In many European countries, fermented foods of plant origins, such as fermented olives, are highly interesting as health-promoting, functional foods that could replace fermented dairy food products for lactose-intolerant humans. The fermented olives have been screened for probiotic lactic acid bacteria. The selected probiotic strains possessed probiotic properties in vitro, including an ability to resist low pH levels and high bile concentrations and an ability to adhere to Caco-2 cells. However, the probiotics did not inhibit the growth of pathogens [41]. The main isolates from green olives were *L. pentosus*, *L. plantarum* and *L. paracasei* and from black olives were *L. pentosus* and *Leuconostoc mesenteroides* [42, 43]. One strain of LAB isolated from kitchen waste of fermented vegetables was identified as *L. delbrueckii*, which inhibited the growth of some pathogens, including *Proteus vulgaris*, *Bacillus subtilis*, *Pseudomonas aeruginosa*, *E. coli*, and *Klebsiella pneumoniae* [44]. Naturally fermented Croatian dry fermented sausage has been screened to find LAB; *L. plantarum* and *L. brevis* were the main species found [45]*.* In addition, in Middle Eastern countries, different fermented foods containing different raw materials, including parboiled dried wheat, garlic, parsley and olives are rich sources of LAB and can be screened for potential probiotics [46].

Several traditional non-dairy fermented beverages are also good sources of probiotics. These beverages are made from a variety of raw materials, including cereals, millets, legumes, fruits and vegetables [47]. These raw materials have been used to make traditional fermented beverages such as *Boza*, *Pozol*, *Bushera*, *Mahewu*, and *Togwa* in several countries in Europe, America and Africa [48]. Cereal grains are a highly nutritional source of protein, carbohydrates, vitamins, minerals and water-soluble fiber, which are materials well-suited to act as prebiotics. Most LAB isolates of *Lactobacillus plantarum* have been isolated from a Turkish traditional fermented drink (*Boza*). These isolates showed antagonistic activity by producing substances (mainly organic acids and hydrogen peroxide) to combat pathogenic bacteria such as *Listeria monocytogenes, Bacillus cereus, B. subtilis, Yersinia enterocolitica, E. coli, Pseudomonas aeruginosa, Salmonella typhimurium* and *Klebsiella pneumonia* [49]. In addition, Oluwajoba et al.[50] isolated probiotic LAB from *Kunu-zaki*, a Nigerian traditional fermented drink made from non-germinated sorghum and millet cereal grains. These LAB species showed promising probiotic properties, including resistance to a pH 3 and to 3 % bile and antimicrobial activity against the referent strains of *Staphylococcus aureus*, *Escherichia coli*, *Pseudomonas aeruginosa* and *Enterococcus faecalis*. These species were identified as *Lactobacillus, Pediococcus* and *Lactococcus* species but were primarily *Lactobacillus* species.

### Probiotic isolates used in humans

For the last two decades, the probiotics that were selected for use in humans originated from the human body, mainly feces or breast milk, or from human foods, which were usually fermented dairy products. The probiotics used in animals, in contrast, often came from animals’ own digestive tract or from probiotic strains originating in human subjects. Probiotic strains can be isolated directly from natural fermented milk products or milk and can then be added as starter cultures for fermentation in products such as cheese, yogurt, and butter. Potential probiotics have been isolated from human sources from different parts of the human body, including the human feces of both healthy adults [51] and breast-fed infants [52], as well as human breast milk [53, 54].

Probiotics isolated from human breast milk have usually been of the *Lactobacillus* genus [54, 55], while probiotics from the feces of healthy human adults and breast-fed infants have been from at least two genera: *Lactobacillus* [55–57] and *Bifidobacterium* [52]. A few studies have reported probiotic *Enterococcus faecalis* found in human feces [58]*. L. salivarius* has been found in human milk and infant feces in individuals of a mother-child pair [59]. *L. rhamnosus* and *L. casei* were isolated from human breast milk and showed resistance to low pH (pH 3), tolerance against a 0.3 % bile concentration and antimicrobial activity against *Escherichia coli*, *Bacillus cereus* and *Staphylococcus aureus* [54]. Another genus of probiotic, *Pediococcus*, which produces a bacteriocin-like substance, can be isolated from healthy human breast milk [60]. Lactobacilli have been isolated from the feces of children aged 4–15 years. One of the 20 lactobacilli isolated from these specimens was identified as *L. pentosus* and possessed basic probiotic properties of acid and -bile tolerance and antimicrobial activity. This isolate also had other probiotic properties, including the abilities to produce and aggregate exopolysaccharides (EPS) and to provide a cholesterol removal effect [61].

### Probiotic isolates used in farm animals

The direct fed microbial (DFM) supplementation concept involves microorganisms mixed with feed to benefit the animals. It is mainly used in the US. In European countries, probiotics have been developed for use mainly in animal production. This supplementation is also based on the administration of one or several live microorganisms, usually yeast or bacteria. In the last two decades, most *Lactobacillus* strains used in humans have also been used as probiotics in animals, but *Bifidobacterium* strains isolated from a human origin were used as probiotics only in humans. Over the past 20 years, the probiotic strains widely used in animals, especially those used in Europe and Japan, are spore forming bacteria of the genus *Bacillus* [22]. Currently, most of the probiotics used in animal farming are lactic acid bacteria (LAB). Sources of probiotics for use in various animal species, including poultry [62], pigs [63] and ruminants [64], are mainly the gastrointestinal (GI) tracts of the same animal species. These probiotics can also be isolated from the feces of different animal species, including chickens [65], pigs [66] and ruminants [67]. Probiotics isolated from an animal species have also been used in another animal species. They may also come from other sources, including fermentation products of plants and animal origin. *B. pumilus* WIT 588 isolated from sea water has been tested in animals and exhibits an ability to inhibit the growth of *Escherichia coli* [68]. *Propionibacterium freudenreichii* isolated from dairy products has been used to reduce enteritis and to improve health in pigs [69]. Several researchers have isolated probiotics from different sources and used in animals as summarized in Table 1.

**Table 1:** Probiotic strains used in farm animals

Probiotic strains	Sources	Identification techniques	Activities	References
*Streptomyces* sp. JD9 (KF878075)	Indigenous and broiler chickens	PCR with universal primers and 16S rRNA gene sequencing	Enhanced broiler production	Latha et al. [70]
*Wickerhamomyces anomalus* LV-6	Broiler chickens	PCR-fingerprinting technique and 26S rRNA gene sequencing	Enhanced broiler production	García-Hernández et al. [71]
*Lactobacillus salivarius* 15 K	Chickens	PCR with specific primers and16S- 23S rRNA gene sequencing	Against *Klebsiella* and *Escherichia coli.*	Bujnakova et al. [72]
*Lactobacillus plantarum* TN8	Indigenous Poultry	PCR with specific primers and 16S rRNA gene sequencing	Imunomodulation in vitro	Ben Salah et al. [73]
*Propionibacterium acidipropionici* LET 105	Laying hens	PCR with universal primers and 16S rRNA gene sequencing	Produced short chain fatty acids (SCFA) and against *Salmonella*	Argañaraz-Martínez et al. [74]
*Lactobacillus plantarum* P-8	Traditional fermented dairy products	PCR with universal primers and 16S rRNA gene sequencing	Imunomodulation in broilers	Wang et al. [75]
*Lactobacillus salivarius* DSPV 001P	Broiler chickens	PCR with universal primers and 16S rRNA gene sequencing	Colonization in intestinal broilers	Blajman et al. [76]
*L. plantarum* (strain P6)*, L. paraplantarum* (strain P25)*, and L. reuteri* (strain P30)	Cows, pigs, chickens, and ducks.	PCR with universal primers and 16S rRNA gene sequencing	Anti-pathogens in vitro	Pringsulaka et al. [77]
*Lactobacillus johnsonii,* *L. salivarius,* *L. murinus,* *L. mucosae,* *L. amylovorus,* *L. mucosae*	Young calves	PCR with universal primers and 16S rRNA gene sequencing	-Anti-pathogens in vitro -Adhesion property	Maldonado et al. [78]
*Lactobacillus reuteri* DDL 19, *Lactobacillus alimentarius* DDL 48, *Enterococcus faecium* DDE 39, and *Bifidobacterium bifidum* DDBA	Goat	PCR with universal primers and 16S rRNA gene sequencing	-Increased milk production and polyunsaturated fatty acid -Antimutagenic activity	Apás et al. [79]
*Bacillus subtilis* KN-42	Weaned pig	PCR with the primers for denaturing gradient gel electrophoresis and 16S rRNA gene sequencing	-Reduced *E. coli* -Increased ADG and FCR improvement	Hu et al. [80]

Giang and co-authors have isolated LAB from different parts of the intestines of healthy fattening pigs [63]. These bacterial strains included *Enterococcus faecium*, *Lactobacillus acidophilus*, *Pediococcus pentosaceus* and *L. plantarum* and were used as probiotic complexes to improve the growth of weaned piglets. Iniguez-Palomares et al. [81] reported *Lactobacillus* strains isolated from the small intestines of piglets; most of these strains were of the *L. salivarius* species. These strains showed promising probiotic properties, including resistance to a pH of 3 and to conjugated porcine bile, auto-aggregation effects and an ability to strongly inhibit the pathogen *E. coli* K88.

LAB species have been isolated from silages in hot and humid weather. They can be used as starter cultures in silages for ruminant. These strains were *Lactobacillus plantarum*, *L. pentosus*, *L. rhamnosus*, *L. buchneri*, *L. rapi*, *Pediococcus pentosaceus* and *P. lolii* [82]. LAB isolated from fecal young calves such as *L. murinus*, *L. johnsonii* and *L. salivarius* had an ability to produce bacteriocin-like activity against pathogens [83].

Fuller reported microbial communities in chicken guts, found amount 29 genera. Each genus is distinguished as being of 3 to 4 species and each species is separated into 3 to 4 subspecies depending on the different mechanisms of the microorganisms. Therefore, the number of types of microorganisms in chicken guts is found to be over 200 species. In addition, wild types of chicken were found to have a greater amount of intestinal microflora than chickens that were commercially raised due to an opportunity for these microbes to receive from hens [2].

Probiotic bacteria widely used in aquatic animals are LAB and *Bacillus* species. Most these probiotic strains are isolated from aquatic animals from their gastrointestinal tracts. Several studies have reported the probiotic isolates, isolated from both fresh water and sea water animals. Diaz et al. reported that *Lactobacillus salivarius* from bottlenose dolphin can inhibit the growth of *Salmonalla* Enteritidis strains that isolated from both marine animals and humans [84]. *Leuconostoc mesenteroides* strains have been isolated from the intestines of fresh water fishes such as snakehead fish [85] and Nile tilapia fish [86]. They showed an ability to inhibit the growth of fish pathogens. *Bacillus pumilus* and *B. clausii* isolated from the guts of fish grouper *Epinephelus coioides* showed beneficial effects, including an ability to inhibit the fish pathogens, growth performance improvements and an immune stimulation [87].

Recently, Munoz-Atienza et al. [88] used probiotic *Leuconostoc cremoris* and *Weissella cibaria* isolated from Atlantic salmon fish and common octopus, respectively to inhibit the marine fish (turbot fish) pathogens successfully and to stimulate a non-specific immune response. These probiotic isolates can be survived in seawater at 18 °C for 7 days and resisted to pH 3 and 10 % (v/v) turbot bile [52]. Sarkono et al. reported that *Lactobacillus paracasei* isolated from eyes shellfish (abalone) showed a resistance to acidic and bile conditions and an ability to inhibit pathogenic bacteria such as *E. coli*, *Bacillus cereus* and *Staphylococcus aureus* [89].

### Selection for probiotics

Selection of probiotics from different sources involves screening for non-pathogenic bacteria, such as LAB, followed by an evaluation of the basic features of these bacteria, including acid and bile tolerance, ability to adhere to gut epithelial cells and ability to combat pathogens in the GI tract. The pathogenic properties of *Lactobacillus salivarius* isolated from human milk have been tested by an evaluation of oral toxicity in mice [90]. However, novel probiotics must be tested for beneficial properties in both in vitro and in vivo models. The novelty of probiotic strains is considered together with safety requirements, which include a complete genome description and annotation, knowledge regarding the transferability of antibiotic resistance, selection of the proper in vivo model, toxicological studies and designation of the target population, as recently described by Kumar et al. [91].

Selection procedures for probiotics isolated from fermented foods usually include testing for probiotic properties such as a tolerance to heat, acid, bile salt and NaCl. The probiotic strains are also tested for antimicrobial activity against pathogens. In addition, other features have been considered when selecting probiotics. These include the production of bile salt hydrolase [92], the production of exopolysaccharides (EPS) [19, 61, 93], which increase the colonization of probiotics in the gut, an ability to inhibit the harmful fecal enzymes of intestinal microflora, including β-glucosidase, β-glucuronidase, tryptophanase and ureas [51, 72], and safety features. Safety features include being non-pathogenic microorganisms [90] and not being able to transfer any antibiotic resistance genes to other bacteria.

Selection of LAB for use as potential probiotics begins by screening for exopolysaccharide (EPS)-producing bacteria. [19, 61, 93]. Then, the selected bacteria are tested for other properties, including resistance to acid and bile, and an ability to combat pathogens in the GI tract. Microbes that are potential probiotics have been isolated from vegetables and traditional dairy fermented foods; these microbes have the basic features just mentioned and represent approximately 24 % of all EPS-producing strains [19]. EPS-producing bacteria can be found in both fermented dairy and non-dairy foods. The EPS-producing activity of these bacteria is strain specific [93].

Probiotics isolated from intestines in both humans and animals have some probiotic properties that are different from those of the probiotics originating from dairy products. The adhesion of these probiotics is one of the most notable differences. Intestinal isolates usually exhibit higher adhesion activity than the dairy isolates. Bunesova and colleagues reported that *Bifidobacterium animalis* isolated from feces showed strong auto-aggregation activity, which is related to adhesion in the GI tract. This activity was not observed in the probiotic strains isolated from dairy products [94]. In addition, the intestinal probiotic isolates are more likely to be resistant to low pH levels and high concentrations of bile than are the probiotics isolated from dairy origins [95]. However, Monteagudo-Mera et al. reported that some *Lactobacillus* strains isolated from cheese were more tolerant to low pH levels and more adherent to CaCo-2 cells than was *Lactobacillus* spp. isolated from human feces. Therefore, probiotics in dairy products may select for strains that can be isolated from these products for a more beneficial use [83].

### Safety properties of probiotics

One of the important safety properties of probiotic microbes is an antibiotic resistant feature. Generally, antimicrobial resistances of probiotic microorganisms are two characteristics: (i) natural or intrinsic resistance, in which case resistance is not transferable; (ii) acquired resistance, usually caused from bacterial mutation or may carry plasmid encoding of antibiotic resistance genes and potentially transferable to other commensal or pathogenic bacteria [96]. Studies of antimicrobial resistance of probiotic microbes have reported depending on isolation sources and antibiotic tested groups. LAB is one of the large groups to select as potential probiotics and also to test for antimicrobial susceptibility. Analyses of antibiotic resistance of probiotics are in both phenotypic and molecular methods. Broad spectrum antibiotics such as tetracycline and chloramphenicol have been detected antibiotic resistant genes as a horizontal gene transfer in LAB probiotics or starters mainly lactobacilli, including *tet*(W), *tet*(M), *tet*(S), *tet*(O),*tet*(Q), *tet*(36), *tet*(Z), *tet*(O/W/32/O/W/O), *tet*(W/O), *tet*(K), *tet*(L) and *cat* gene, respectively [97]. LAB mostly lactobacilli isolated from various sources, including GI tracts of animals such as chickens [98], dogs [99] and wild boars [100], human feces [101], fermented food products [29, 102] and fermented milk products. Njage et al. have been reported that they can be resisted to tetracycline that they may acquire the resistance gene from other bacteria [103].

### Isolation and identification of probiotics

An initial isolation of probiotic LAB from uncommon sources or non-intestinal sources is cultivation using a high nutritional medium which is different or modification from conventional de Man, Rgosa and Sharp (MRS) medium. For example, LAB isolated from paddy rice silage, crop and silage fermentation have been cultivated using medium consisting mainly glucose, yeast extract and peptone (GYP) [104, 105]. LAB isolated from soil (rhizospheres of fruit trees and soil around animal farms) was cultivated successfully using GYP plus BM medium [17, 18]. BM medium containing the most of tomato juices, peptone, liver extracts and glucose is used to cultivate malolactic-producing LAB such as *Oenococcus oeni* from red wine- making in Japan [106, 107].

LAB screening from various parts of GI tracts of animals is also used a modified MRS medium. In this case, high producing lactic acid of LAB isolates that are usually found in the GI tracts of animals may require some substrates for suitable growths such as pH conditions and nutrients. LAB has been isolated from GI tracts of animals using a modified MRS medium by adding 0.3–1 % (w/v) CaCO_3_. This medium has been used to isolate LAB successfully in various animals, including chickens [62, 98], cattle [108] and dogs [99]. The medium has also been used to cultivate LAB from traditional fermented foods which were made from various raw materials such as fish (*Pla-chom*) [102] and beef (*Mum*) [29]. LAB was cultivated from fermented foods in acidic conditions such as a traditional pickle food using medium consisting the most of glucose, yeast extract and peptone and 0.5 % (w/v) CaCO_3_ [105].

LAB species were isolated from an air surrounding sourdough and bakery room productions. Identification of these LAB using molecular methods depends on culture-dependent assays that they were cultivated using MRS-5, a modified version of high nutritional MRS medium [109], for a sample enrichment that these LAB were successfully growths [27]. Thus, screening of probiotic LAB from uncommon sources, such as soil and air can be succeeded using accumulation methods that they are cultivated using high nutrition modified MRS medium such as MRS-5 and GYP plus BM medium under anaerobic conditions.

Screening of some intestinal LAB probiotic and *Bifidobacteria* are also not easy using the best anaerobic culture methods because a large complex bacterial community inhabiting the GI tract and many species of the microbes have never been cultivated under laboratory environments. Indigenous microbiota in intestinal sources in both humans and animals are identified by two main methods. The first one, a culture-based method, is observed phenotype characteristics including biochemical, physiological and morphological tests in accordance with Bergey’s Manual. The culture-based method associated with biological molecular technique is a way for more effective identifications. The second one, a culture-independent method is considered as alternative techniques to investigate the large proportion of the both cultured and uncultured bacteria in GI tracts. These methods are developed to discriminate between species of bacteria from the difference of their DNA fragments in band profiles, such as the PCR in Denaturing Gradient Gel Electrophoresis (PCR-DGGE) and the PCR in Temperature Gradient Gel Electrophoresis (PCR-TGGE). The fluorescence in situ hybridization (FISH) technique was also used to detect the uncultured bacteria [110]. Biochemical testing of LAB identification is widely used by carbohydrate fermentation patterns such as the API (API system, Biomerieux, France). Biological molecular methods have been increasingly used and there are many methods, including a DNA base composition (mol % of guanine plus cytosine), a DNA homology accompanied with polymerase chain reaction (PCR) technique and DNA sequencing using 16S rRNA gene region. 16S rRNA gene sequencing is developed to simplify sequences using species-specific PCR primers that targeted some regions of lactobacilli such as the 16S – 23S rRNA spacer region [111], the shuttle cloning vector to extract the bacterial plasmids [112], internal transcribed spacer PCR (ITS-PCR) [113] and amplifications with amplified ribosomal DNA restriction analysis (ARDRA) [114]. Fingerprinting techniques are the methods to differentiate the microbial community at strain levels. These methods have been developed to discriminate strains of LAB, especially lactobacilli, including the ribotyping [115], randomly amplified polymorphic DNA (RAPD)-PCR [116], amplified fragment length polymorphism (AFLP) [117], plasmid profiling [118] and pulsed-field gel electrophoresis (PFGE) [112]. A PFGE technique is high discriminatory power using the specific restriction enzyme of the genomic DNA of bacteria. A pyrosequencing technique with specific primers [119] is also useful methods to differentiate these probiotic species.

## Conclusions

Alternative sources of probiotics, such as non-dairy fermented food products, present an advantage in the search for new probiotic strains. Increasingly, these probiotic sources are being selected for use in people who are lactose intolerant. The selection of probiotics from different sources involves screening for non-pathogenic microbes followed by an evaluation of basic properties, including acid and bile tolerance, an ability to adhere to gut epithelial cells, an ability to combat against pathogens in the GI tract, and the safety-enhancing property of an inability to transfer any antibiotic resistance genes to other bacteria. Selected probiotics isolated from intestinal sources in both humans and animals are identified using molecular methods by two main methods: culture-based and culture-independent methods. The culture-independent method is considered as alternative techniques to investigate the large proportion of the both cultured and uncultured bacteria in the GI tract.

## Abbreviations

CFU, colony forming unit; DFM, direct fed microbial; EPS, exopolysaccharides; FISH, fluorescence in situ hybridization; GI, gastrointestinal; GYP, glucose, yeast extract and peptone; LAB, lactic acid bacteria; MRS, de Man, Rogosa and Sharp; PCR-DGGE, PCR in denaturing gradient Gel electrophoresis; PCR-TGGE, PCR in temperature gradient Gel electrophoresis; PFGE, pulsed-field gel electrophoresis; RAPD, randomly amplified polymorphic DNA
